# Health literate-sensitive shared decision-making in maternity care: needs for support among maternity care professionals in the Netherlands

**DOI:** 10.1186/s12884-023-05915-9

**Published:** 2023-08-21

**Authors:** Laxsini Murugesu, Olga C. Damman, Danielle R. M. Timmermans, Sanne de Wit, Marianne Nieuwenhuijze, Ellen M. A. Smets, Mirjam P. Fransen

**Affiliations:** 1 Amsterdam UMC Location University of Amsterdam, Public and Occupational Health, Amsterdam, the Netherlands; 2 Amsterdam Public Health Research Institute, Quality of Care, Amsterdam, the Netherlands; 3 Amsterdam UMC Location Vrije Universiteit Amsterdam, Public and Occupational Health, Amsterdam, the Netherlands; 4 Research Centre for Midwifery Science, Zuyd University, Universiteitssingel 60, Maastricht, 6229 ER the Netherlands; 5 CAPHRI, Maastricht University, Maastricht, the Netherlands; 6 Amsterdam UMC Location University of Amsterdam, Medical Psychology, Amsterdam, the Netherlands

**Keywords:** Shared decision-making, Health literacy, Needs assessment, Maternity care professionals

## Abstract

**Background:**

Shared decision-making (SDM) in maternity care is challenging when clients have insufficient health literacy (HL) skills. This study gained insight in how professionals apply HL-sensitive SDM in Dutch maternity care and their needs for support therein.

**Methods:**

Maternity care professionals (*n* = 30) completed a survey on SDM and the role of HL. Midwives (*n* = 13) were observed during simulated conversations discussing pain relief options and interviewed afterwards. The client-actors were instructed to portrait specific inadequate HL skills. Observation items focused on adapting communication to HL, and SDM (OPTION-5).

**Results:**

In the survey, professionals indicated experiencing most challenges when estimating clients’ information comprehension. Observations showed that most midwives created choice awareness and informed clients about options, whereas exploring preferences and actual decision-making together with clients were observed less frequently. Their perceived HL-related obstacles and needs for support related to clients’ information comprehension. In the interviews, midwives reported putting much effort into explaining available options in maternity care, but also that decisions about pain relief are often postponed until the moment of labour.

**Conclusion:**

Professionals’ self-reported needs focus on clients’ information comprehension. However, observations indicate that it is not the stage of informing, but rather value clarification and actual decision-making that need improvement in HL-sensitive SDM.

**Supplementary Information:**

The online version contains supplementary material available at 10.1186/s12884-023-05915-9.

## Background

Maternity care involves decisions for which multiple equivalent options are available, and for which clients are encouraged to seek information beforehand and to express their preferences to their care provider. Shared decision-making (SDM), in which professionals inform clients about available options and encourage them to share their values and preferences, to further discuss treatment options and choose an optimal treatment plan [1], is increasingly being seen as the optimal decision-making model in maternity care [2]. Studies on SDM in maternity care showed that SDM in maternity care reduces clients’ decisional conflict and decisional regret, and contributes to a more positive reflection upon their childbirth [3].

While SDM in itself can be challenging for both health professionals and clients, it requires specific attention to adapt SDM to the health literacy (HL) skills of clients [4]. HL is generally defined as skills to access, appraise, understand and apply information to make informed decisions, as well as numeracy skills to use and interpret probability information [5]. We previously specified HL skills needed for decision-making during pregnancy and labor from the perspective of clients [6], along with the existing framework of McCaffery and colleagues for SDM among patients with lower literacy [7]. Within different stages of decision-making, we found that maternity care clients with low functional HL levels mainly perceived difficulties in finding reliable information about pregnancy and labor, understanding probabilistic information, constructing preferences based on benefit/harm information, preparing for regular check-ups, and coping with changing circumstances and uncertainties around pregnancy and labor. Primigravidas experienced similar difficulties as clients with low functional HL levels, presumably having problems finding, understanding and using information in this context because of their new role as first time parent [6].

Adapting communication to HL levels is considered important in supporting patients to actively participate in decision-making across HL levels [4, 8]. For HL-sensitive communication in general, it has been argued that provider actions such as checking comprehension, providing and explaining additional written information, explaining terms and abbreviations, speaking slowly, and encouraging patients to ask questions are important [9]. For SDM specifically, attempts have been made to simplify Patient Decision Aids and other tools, e.g., by improving readability or reducing the cognitive effort needed to process the information [4, 10]. However, these interventions are quite narrow in their focus on functional HL skills needed to understand and use information materials. Less attention has been given to how professionals can support individuals in the consultation room to apply the full range of HL skills needed for SDM [4, 8].

Specifically in the context of maternity care, research on SDM with clients with low HL levels is scarce and merely focused on information provision or improving clients’ HL levels in maternity care [11–15]. Only a few studies in maternity care explored HL-related problems in communication beyond information provision, including SDM, all performed in Australia [16–18]. One qualitative study among professionals in maternity care indicated that they perceived non-attendance at appointments, time constraints, and high workload as barriers to optimally inform clients with limited HL [16]. Also, professionals appeared to often overlook HL indicators such as poor reading skills, particularly when time-pressed [16]. Another observational and interview study showed that midwives’ attempts to tailor health information to individual needs were frequently based on incomplete information about clients' HL level [17]. This finding corresponds to findings from a recent survey study, showing that 77% of midwives reported paying limited attention to assessing clients’ HL level [18]. Taken together, these findings suggest that low HL is a complex problem during maternity care consultations and that more research is needed into HL-sensitive SDM practices and professionals’ needs for support.

This study aimed to gain insight in how professionals apply HL-sensitive SDM in maternity care and their needs for support, thereby addressing the following research questions:

- RQ1. How do maternity care professionals apply HL-sensitive SDM?
- RQ2. What problems do these professionals encounter when they apply HL-sensitive SDM?
- RQ3. What support do these professionals need to apply HL-sensitive SDM?

## Methods

### General study design, population and recruitment

This study consisted of three parts: (1) an online survey study among diverse professionals in maternity care (December 2020); (2) an observational study of online simulated consultations of midwives (September – December 2021); and (3) additional interviews with the midwives who participated in the observational study were held to gain further insight into midwives’ behavior during the simulated consultations and daily SDM practices (October–December 2021).

Participants in the survey study consisted of obstetricians, obstetric residents, midwives, physician assistants, nurses, professionals in youth health care, and paediatricians. They were recruited through existing national maternal care networks and professional associations by direct email invitations. Furthermore, this invitation was shared through newsletters of a regional maternity care network and posts on websites and LinkedIn. For the simulated observational study and additional interviews, we chose a homogenous group of professionals comprised of midwives in primary and secondary care settings, to explore how midwives apply SDM among low-HL clients. Dutch maternity care is organised in two echelons, midwife-led care and obstetrician-led care, with professionals in these echelons working alongside and complementary to each other [19]. Midwives in the Netherlands work independently in the community in group practices (midwife-led care), or in hospitals (obstetrician-led care) under supervision of an obstetrician. Midwives were chosen for the observations and additional interviews, since they are the main professionals in the Dutch perinatal care landscape, with 85% of all pregnant women starting their antenatal care with primary care midwives [19]. Recruitment was done through newsletters of several maternal care networks and via individual practices. The contact details of the researcher (LM) were provided for midwives to volunteer or respond for more information. Written informed consent was obtained prior to the survey, and prior to the observations for video recordings in Microsoft teams and for audio recording of the interview via an audio-recording device. The video- and audio recordings were stored with respondents’ anonymized ID number in a secured file only accessible to the researchers MF, SdW and LM.

### Online survey study

#### Design

The survey was administered in Castor EDC. Professionals who were interested received a personal link to complete the survey. The survey explored how maternity care professionals apply SDM with low-HL clients (RQ1), what problems professionals encounter (RQ2), and what support needs professionals have when they apply SDM with low-HL clients (RQ3). The following *background characteristics* were asked about: age, sex, profession, and years of work experience. A brief explanation of the SDM steps was given in the introduction and a working definition of HL was given at the beginning of the survey. For RQ1, we used the specific case of the decision between various pain relief options. We chose pain relief during labour because, based on the Dutch National Care Standard Integrative Maternity Care, midwives and obstetricians in the Netherlands are obliged to discuss medical and non-medical pain relieving options during pregnancy with clients and it is seen as a preference–sensitive decision [20]. Pain relieving medication include epidural analgesia, remifentanil, pethidine injection and nitrous oxide. Decisions on which pain relief option to choose relate to the place chosen for birth and (clinical) circumstances during the birth itself. According to the Standard Operating Procedure (SOP) by the Dutch Health Care Inspectorate, remifentanil is only recommended as an alternative to epidural analgesia when contraindicated and under requirements such as education for healthcare providers, the procedure to obtain informed consent, maternal monitoring requirements, preparation for the application of the method, treatment for complications and documentation [21]. Furthermore, according to the Dutch National Care Standard Integrative Maternity Care, it is recommended to inform clients about pain relief options including their benefits and harms before 34 weeks gestation and to record preferences in a birth plan [20]. Professionals’ experienced problems (RQ2) and needs for support (RQ3) were first explored for SDM in maternity care in general as a prelude to the HL-specific questions that followed.

#### Procedure and measures

The survey started with how often professionals *apply SDM* regarding pain relief options specifically on a 5-point Likert scale ranging from ‘never’ to ‘always’. Professionals who generally do not counsel or provide care around pain relief during birth were directed to the questions regarding SDM in general, and low-HL clients specifically (explained below).

*Most and least frequently experienced challenges in applying SDM* were assessed through statements on SDM (see Supplementary file 1: survey) that participants had to rank from ‘most difficult’ to ‘least difficult’ and vice versa. In an additional open question, professionals were asked to explain what other challenges they experienced when applying SDM concerning pain relief options during birth, in general, and specifically when counselling low-HL clients.

*Current use of supporting tools* was measured by asking about which tools they used (predefined options are presented in Supplementary file 1: survey). An open question asked about reasons for using these tools and what these tools might lack. Professionals who indicated not using any tools were asked for clarification.

*Needs for support in SDM in general, about pain relief options, and with low-HL clients* were assessed as follows: “To what extent do you need support for SDM *in general/concerning pain relief options/ with low-HL clients*?” on a 5-point Likert scale ranging from ‘no need for support at all’ to ‘very much in need of support’. In an open question, we assessed in which format they preferred to receive support.

#### Data analysis

Descriptive data were extracted from the survey data using SPSS version 26.0 and open questions were examined qualitatively to inventory additional tools and needs for support.

### Observation study of online simulated consultations

#### Design

All midwives were observed in two simulated consultations concerning pain relief during birth with two actors each portraying clients (30-week pregnant primiparas) with varying HL skills. The conversations were held in a fixed order, one after the other. All midwives started with actor-client 1, and after the first round, they held a simulated consultation with actor-client 2.The topic of pain relief was again chosen as case example since pain relief is, in principle, discussed with all pregnant women in Dutch maternity care [20]. According to the Dutch national Care Standard Integrative Maternity Care, it is relevant to discuss the options *before* birth, as the decision is related to the place chosen for birth and (clinical) circumstances during the birth itself. Since pain relief is a preference-sensitive decision with multiple medically reasonable options, SDM is considered particularly relevant.

#### Portrayal of low HL skills in simulated consultations

The HL skills that the actors had to demonstrate were systematically selected from our previously developed framework [5, 6]. Instructions for actors were prepared in collaboration with maternity care professionals, client advocates, communication researchers, and a trainer in SDM, all of whom were part of the project team. Actor-client 1 was instructed to have difficulties selecting and appraising information, understanding the harms and benefits of options, understanding the likelihood of harms occurring, and carrying out basic calculations. Actor-client 2 was instructed to have difficulties finding sources of information about pregnancy and birth, preparing for a consultation, interpreting pregnancy-related terminology, understanding that involvement and choice are possible for her, and constructing her preference.

#### Procedure and measures

Midwives were instructed to mirror the behaviour of their daily practice in regard to discussing pain relief, to use tools if needed, and to take a maximum of 20 min. Due to COVID-19, the simulated consultations with midwives were held online. The observations were video-recorded in Microsoft Teams and scored by two raters (a clinical resident, and a SDM trainer and coach) both experienced in observational coding. The Observing Patient Involvement (OPTION-5) tool was used to score midwives’ communicative behaviour associated with SDM during the different SDM stages, namely creating choice awareness, information provision, value clarification, and decision-making [22]. Items were rated on a 0–4 scale. Five self-constructed items based on an operational definition of teach-back (i.e. asking patients to restate given information) [23] were used to measure HL-sensitive communication (see Supplementary file 2 for observational protocol). These included ‘chunk information and check understanding’, use of plain language, use of tools or non-verbal acts (i.e. conversational hand or facial gestures [24]), and application of teach-back. Items were rated as ‘not done at all’, ‘done’, ‘done well’, or ‘not applicable’. A further four self-constructed items were used to assess in which format (verbally or numerically) probability information was communicated. Risk communication was scored on the basis of criteria for adequate risk communication, i.e. using frequencies or percentages as consistent formats, keeping the denominator constant when two or more probabilities need to be compared, and using evaluative labels to improve understanding [25].

#### Data analysis

The scripts for actors and the observation protocol were explained to both raters, who double-coded a total of eight simulated consultations randomly selected across all observations. The observation protocol was finalized by comparing scores and discussing discrepancies after every two double-coded consultations in regular meetings. After finalizing the observation protocol, raters independently scored remaining observations. Additional meetings were held to moderate coding of the remaining observations. After a set of four coded consultations, interrater agreement was calculated for all items separately and for the overall OPTION-5 and HL-SDM scores [10]. The overall intraclass correlation (ICC) scores for OPTION-5 (ICC = 0.7) and HL-SDM (ICC = 0.7) items were considered ‘good’^1^. The overall ICC’s scores and the average weighted Kappa’s for each item separately can be found in Supplementary file 3.

### Additional interviews

#### Design and procedure

Midwives who participated in simulated observations were interviewed online due to COVID-19 via Microsoft Teams approximately five days to two weeks later. Written informed consent was gained for audio-recording. The aim of the interviews was to explore the midwives’ perspective on how they generally apply SDM with low-HL clients (RQ1), which problems they generally encounter when they apply SDM with low-HL clients (RQ2), and their perceived needs for support when applying SDM with low-HL clients (RQ 3).

#### Measures

The interview guide covered topics related to SDM in daily practice, needs for support in SDM, and a reflection of the simulated consultations. For the latter, midwives’ response to actors’ inadequate HL skills were shown during the interview to further explore midwives’ SDM practices. The following background variables were assessed orally: age, sex, and years of work experience.

#### Data analysis

**Fig. 1:**
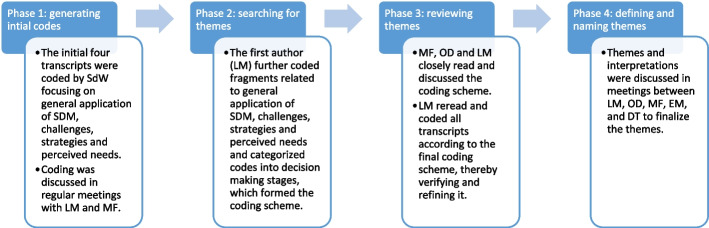
Phases of coding and analysis

The audio-recordings of the interviews were literally transcribed and deleted afterwards. We reached information completeness within the group op observed midwives. The transcripts were then thematically analysed by LM and SdW using MAXQDA2020, as illustrated in Fig. 1 [27]. Themes regarding applying SDM, challenges, strategies used, and perceived needs for support were initially categorised according to decision-making stages [6, 7]. The themes were discussed in regular meetings between the authors.

## Results

### Response and background characteristics of survey, observations and interviews

A total of 30 professionals completed the survey. Thirteen midwives participated in the observations and additional interviews (Table 1). In total, 87% of the survey respondents and all midwives participating in the survey, observations and interview were female, which is representative for midwives working in the Netherlands [28].

**Table 1:** Demographics respondents survey (*n* = 30), observations and interviews (*n* = 13)

**Survey (*n*** **= 30)**	Mean (SD; range)	N (%)
**Age in years**	46 (10; 27–60)	
**Sex**		
Male		4 (13%)
Female		26 (87%)
**Years of work experience**	14 (10; 3–36)	
**Profession**		
Obstetrician		16 (53%)
Midwife		5 (17%)
Obstetric resident		4 (13%)
Other (a hospital based midwife, a physician assistant, a project leader and two nurses)		5 (17%)
**Observations and interviews (*n*** **= 13 midwives)**	**Mean (SD; range)**	**N (%)**
Age in years*	48 (9; 37–62)	
**Sex**		
Female		13 (100%)
Years of work experience *	17 (10; 4–33)	
**Hospital based midwives**		6
**Community midwives**		7

^*^5 missing values

### Outcomes of online survey study

#### Application of SDM

**Fig. 2:**
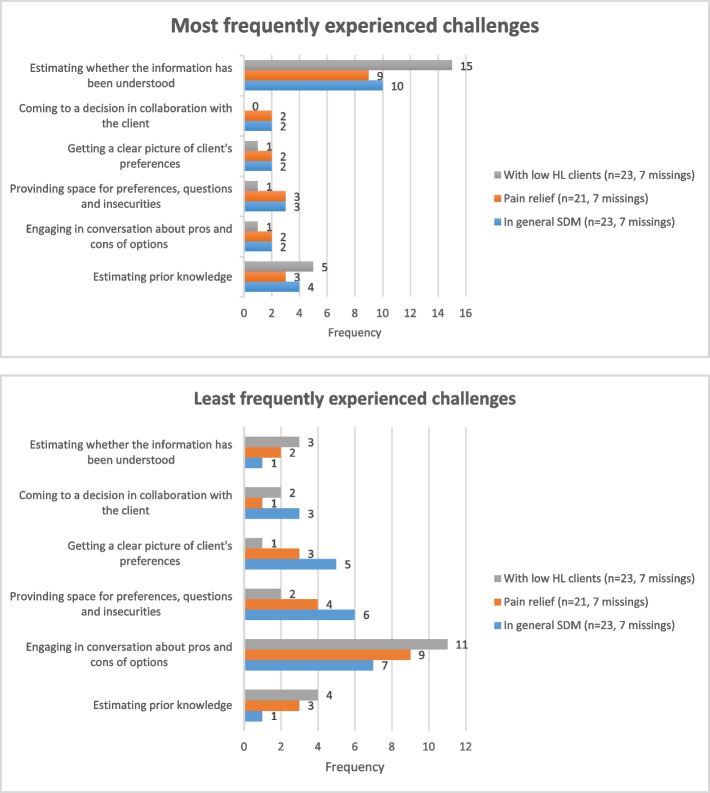
Most and least frequently experienced challenges in applying SDM (missing data were not related to specific professions)

Half of the respondents (*n* = 15/30) reported often or always applying SDM when discussing pain relief options, while a few (*n* = 3/30) reported never using SDM for this specific case example. Figure 2 shows that the problem most often encountered was estimating clients’ comprehension of the provided information (10/23 and 15/23, respectively). Providing information about the benefits and harms of options was reported to be the least problematic SDM step (*n* = 7/23 for SDM in general vs. *n* = 11/21 with low-HL clients) (Fig. 2).

Most professionals reported not using tools to support general SDM (15 out of 23) nor among lower-HL clients (14 out of 23), whereas they did use tools when discussing pain relief options (14 out of 23). Reasons for not using tools were unawareness of their existence (*n* = 4), unavailability of tools (*n* = 5), or not experiencing a need to (*n* = 2).

Professionals reported using tools to support SDM in general most frequently, including option grids (*n* = 9), online decision aids (*n* = 8), and pamphlets (*n* = 9). Among lower-HL clients specifically, professionals reported using tools including online decision aids (*n* = 3) and translation tools (*n* = 4). Additionally, about half of the professionals (12 out of 23) reported using the teach-back method as a communication skill, and aligning the information to the HL level of the client (*n* = 10).

#### Needs for support in SDM

For SDM in general, more than half of the respondents indicated no clear need for additional support (10 out of 14, 16 missing values). However, in the context of lower-HL clients, 5 out of 14 (16 missing values) respondents experienced some need for support and three respondents experienced a high need for additional support. Among the nine respondents not using tools in HL-sensitive SDM, five reported some need for additional support and two indicated a high need. In response to open-ended questions, respondents reported needs for decision support aids and/or option grids in multiple languages (*n* = 7), training in SDM (*n* = 3), videos (*n* = 1), a pregnancy phone application (*n* = 1), and algorithms to predict individual risks (*n* = 1).

### Observation study of online simulated consultations

#### OPTION-5 scores

In total, 26 video-recordings of 13 midwives were scored. Midwives scored highest on OPTION-5 items for creating choice awareness and providing information about pain relief options during birth (see Table 2). However, midwives scored relatively low on reassuring clients that they would receive support in being informed and on supporting clients to voice and explore personal preferences in pain relief options. Relatively low scores were also obtained for making an effort to integrate the client’s preference in the provisional decision about pain relief options or arriving at a provisional decision through collaboration and discussion.

**Table 2:** Outcomes of observation items focused on adapting communication to HL, and OPTION-5 (*n* = 13)

	**Actor-client 1**	**Actor-client 2**	**Total**
	Mean* (SD; range) **Actor-client 1**	Mean* (SD; range) **Actor-client 2**	**Mean (SD; range)**
**OPTION-5 items**			
Creating choice awareness	3 (0.9; 2–4)	3 (0.7; 2–4)	3 (0.8; 2–4)
Reassuring the client they would receive support in being informed	1 (1.7; 0–4)	2 (1.6; 0–4)	2 (1.6; 0–4)
Giving information about options	3 (0.5; 2–3)	3 (0.7; 1–3)	3 (0.6; 1–3)
Voice and explore personal preferences	1 (1.1; 0–4)	1 (1.6; 0–4)	1 (1.0; 0–4)
Integrating patient’s preferences as provisional decisions	1 (1.5; 0–4)	1 (1.6; 0–4)	1 (1.5; 0–4)
OPTION-5 total**	9 (3.2; 5–15)	9 (3; 4–16)	9 (3.1; 4–16)
**Health Literacy items**	**n (%)**	**n (%)**	**n Total (%)**
Chunk and check			
Not observed	10; 77%	11; 85%	21; 81%
Done	3; 23%	2; 15%	5; 19%
Using plain language			
Not observed	8; 62%	5; 39%	13; 50%
Done	5; 39%	5; 39%	10; 39%
Done well		3; 23%	3; 12%
Using tools or non-verbal acts to support understanding			
Not observed	5; 39%	3; 23%	8; 31%
Done	7; 54%	10; 77%	17; 65%
Done well	1; 8%		1; 4%
Applying teach-back			
Not observed	12; 92%	12; 92%	24; 92%
Done	1; 8%	1; 8%	2; 8%

^*^ = rounded up

^**^OPTION-5 total scale ranges from 0 to 20; OPTION-5 items scale ranges from 0 to 4

#### HL-sensitive communication

Midwives gained sufficient scores (‘done’ or ‘done well’) for using plain language and tools or non-verbal acts to support understanding (see Table 2). The majority of the midwives (65%) used conversational hand gestures to improve understanding among the actor-clients, for example about dilation, or referred the client to websites or informational material. Two midwives used the teach-back method during the conversations.

#### Communication of outcome probabilities of options

Ten out of thirteen midwives discussed probability information with actor-clients. Midwives discussed probability information with both actor-clients (in seven conversations with client 1 and in six conversations with client 2). They mainly discussed possible harms of having epidural analgesia (side-effects such as fever, headache, risk of paralysis, and ‘other risks’) and remifentanyl (risks of having breathing difficulties and having ‘other risks’). Probabilities of becoming paralysed as a result of epidural analgesia were only discussed with actor-client 2, mainly in reaction to her questions about having epidural analgesia (which was part of this actor-client’s script). In seven out of the ten conversations where probabilities were discussed, probability information was provided using verbal labels (e.g. ‘small risks’). In four out of the ten conversations, mainly percentages were provided in addition to the verbal label (e.g. ‘a small risk, namely x%’). Two midwives used framing to explain the side-effects of pain relief options, e.g. if × 2 of 100 clients suffer from this harm, it means that 98 out of 100 clients do not suffer.

### Insights obtained from additional interviews

A total of five themes were identified from the interview data. Table 3 presents these themes.

**Table 3:** Themes and illustrative quotes from interviews among midwives

Theme 1: lack of knowledge on what the SDM model entails in essence	*Quote 1, respondent 101: “My task is to inform them about the options and, for each decision they make, to be convinced that they fully aware of what they have decided and to support them in this.”*
Theme 2: reluctance among midwives to discuss preferences towards options in advance, due to uncertainty around birth	*Quote 2, respondent 105: “…but ultimately, it is an experience at that moment of labour, when we actually see how things are going. You can’t prepare yourself for every possible situation and you can’t make decisions during the pregnancy about what you would like. That’s just not possible.”*
Theme 3: midwives apply various strategies mainly focused on supporting clients’ functional HL skills	*Quote 3, respondent 103: I find it really difficult to estimate their skills, really hard. In the beginning you ask about the level of education for their job, but of course that doesn’t tell you the whole story. You talk to someone, which gives you an idea: they understand this or they don’t understand that, it’s difficult, and I’d prefer to receive some tips and tricks for that; if it’s possible to learn something about that, I’d be interested*
Theme 4: communication about probability information is simplified	*Quote 4, respondent 209: “Not consciously, but naturally, not like: I’m not going to tell you that, but the conversation just tends to proceed in a simpler manner. As long as they’ve understood the most important pros and cons and the risks you have to inform them about. And going into things in depth and giving background information, well it sometimes comes across as though they don’t feel the need for it.”*
Theme 5: levelling between the different SDM steps is needed to support value clarification among clients	*Quote 5, respondent 110: “That’s difficult sometimes, because those people don’t always understand everything very well, and sometimes they’ve decided long beforehand what they want and what they don’t want, even though they don’t give any good reason at all for it.”*
Theme 5: levelling between the different SDM steps is needed to support value clarification among clients	*Quote 6, respondent 101:**** “****It all depends on what’s the most urgent for them at that moment. Of course I can tell them a whole story about giving birth smoothly, but if someone doesn’t know if they’ll still be in work the next week, or how they’re going to arrange childcare for the other children, do you see? Then her mind’s just occupied with other things, and the baby will arrive anyway, one way or another.”*
Theme 5: levelling between the different SDM steps is needed to support value clarification among clients	*Quote 7, respondent 108: “But OK, then I get a rough idea after 1 or 2 or 3 consultations. So then I say, don’t worry, we’ll just see how things go, I know you well enough and then we’ll discuss things together at that moment, but I think you’re leaning more in this direction. You still make your assumptions, and you make a summary and draw conclusions. And quite often they’ll say something like: oh yes, we’ll do that, that’s what we’ll do.”*

#### Theme 1: lack of knowledge among some midwives on what the SDM model entails in essence

Midwives in our study seemed to confuse informed consent or informed decision-making with SDM. For example, asking for agreement or consent before conducting a medical procedure such as placing a foetal scalp electrode was mentioned as part of SDM. Some midwives considered their role in SDM as merely informing their clients about the options available in order for clients to make their own decisions, which is described as’informed decision-making’ in many conceptual models (quote 1, Table 3). Two midwives explicitly mentioned that they did not yet have enough experience in counselling or general SDM.

#### Theme 2: reluctance among midwives to discuss preferences towards options in advance, due to uncertainty around birth

The participating midwives emphasised that decisions cannot be made beforehand and depend on the medical situation and context during the birth itself, for example the availability of an anaesthetist in the case of pain relief options, medical uncertainties around birth, or continuity of professionals (e.g. changing shifts of professionals) (quote 2, Table 3). Some midwives mentioned leaving the decision open and deciding at the moment itself. Other midwives mentioned that it is important to discuss options in advance of birth, because clients are unable to obtain or process information when giving birth.

#### Theme 3: strategies applied by midwives mainly focus on supporting clients’ functional HL skills

The challenges reported by midwives, strategies they said they use to tackle these challenges, and needs for support mainly focused on informing clients (Table 4). Although midwives rarely mentioned using decision aids, they did mention needs for support in using such tools or training on HL-sensitive SDM (quote 3, Table 3). Interestingly, some midwives mentioned that it is *easier* for them to explain information to low HL clients, because these clients more easily accept information that is given to them than clients with higher HL levels.

**Table 4:** Perceived challenges, strategies and needs for support focused on clients’ functional HL skills reported in interviews by midwives

Challenges related to low HL level	Strategies applied by midwives to tackle experienced challenges	Additional needs reported by midwives to tackle experienced challenges
Clients being unprepared for consultations	Send information in advance	
Clients use unreliable sources	Provide factual information including reliable social media channels	
Clients underestimate birth pain	Explore existing knowledge and expectations	
Clients’ lack of general knowledge about pregnancy and birth		More time to explain basic knowledge
Clients’ unawareness of choice	Actively mention choice and available options	
Clients do not understand information about options or do not actively ask questions	• Chunk information • Repeat information (verbally and in writing) • Draw or depict information in body language • Read information together with client • Advise reading information with significant others • Advise recording the question on the phone • Teach-back	• More time needed for explanations • Training or guidelines to gauge clients’ HL level • Icon arrays • Easily accessible visual aids • Consultation cards • Option grids
Clients’ inadequate language proficiency		• Informational materials in different languages • Translating aids
Clients ask for professionals’ opinion	Use daily examples to put information into context, such as ‘how do you deal with pain in other situations?’	

#### Theme 4: communication about probability information is simplified

Midwives mentioned explaining only the most common outcomes to low-HL clients (quote 4, Table 3). For example, one midwife reported that she uses her professional experience to explain how often she experienced a certain harm in practice. In general, with low-HL clients, midwives mentioned using absolute numbers, verbal terms, or icon arrays to simplify probability information.

#### Theme 5: levelling between the different SDM steps to support value clarification among clients

Midwives mentioned that clients have often already decided what they want prior to the consultation (quote 5, Table 3). In these cases, midwives reported actively inquiring after their clients’ reasons for choosing a certain option. Some midwives mentioned that low-HL clients do not pay sufficient attention to decisions they need to make during their pregnancy, in the case of disadvantageous social-economic context factors (quote 6, Table 3). In these cases, midwives mentioned steering clients towards a decision they think is best, based on previous conversations with these clients (quote 7, Table 3).

## Discussion

### Summary of main findings

This study gained insight in how professionals in the Netherlands apply health literate-sensitive shared decision-making (SDM) in maternity care and in professionals’ needs for support. Both the survey among diverse maternity care professionals and observations among midwives indicated that professionals experienced relatively few problems in giving information about benefits and harms of options and creating choice awareness. Professionals said they support clients in information comprehension, for example by using plain language and not so much through strategies such as the teach-back method or existing tools to support SDM. However, observations suggested that supporting clients in exploring preferences and making decisions is challenging for midwives and they hardly used tools to support them in this. Nevertheless, professionals in both the survey as well as interviews did not express a need for support in value clarification and making decisions, but rather indicated a need for tools that actually support clients’ comprehension of information.

### Discussion of main findings

Professionals in this study appeared to mainly focus on informing clients, and they themselves did not report explicit problems related to the other SDM steps, such as value clarification (i.e. in the OPTION scale: ‘Voice and explore personal preferences’). This emphasis on informing clients is in line with previous results in the maternity care context [29], and has also been observed in other contexts among low-HL patients [30]. For example, a previous observational study among low-HL patients in the palliative phase of their disease also indicated lowest scores for value clarification in SDM [31]. Although clear information provision is a prerequisite for effective SDM, it is not sufficient. Value clarification and subsequent deliberation, where patients are coached to trade-off benefits against harms, are also crucial in reaching a shared decision [4, 32].

One reason why professionals may be primarily focused on information provision, at least in relation to discussions about pain relief options, may be that the Dutch National Care Standards for Midwifery also emphasises information provision rather than further decision support. These standards mention the importance of SDM when deciding about pain relief options, but also that the final decision is primarily made by the client giving birth. This seems to contrast to some extent with standard SDM models, which describe that professionals decide in collaboration with patients after thoroughly weighing benefits and harms of all options together. Another reason could be that professionals were unaware of what SDM actually involves. Related to this is our finding that some midwives seemed to confuse SDM with other models, such as informed consent and informed decision-making. This has also been demonstrated previously among other maternity care professionals in Australia [33, 34]. Training for students and professionals thus seems essential for providing the knowledge and skills required for SDM [35].

The midwives interviewed in this study also mentioned often postponing decisions related to pain relief options until the moment of labour. The observations and interviews indicated that the midwives seemed comfortable discussing pain relief options antenatally, however they seemed to be reluctant to discuss preferences and decisions about pain relief options in advance, due to changing circumstances during birth. While this is understandable from the perspective of professionals, a previous qualitative study in the U.K. concluded that it would be more beneficial to concentrate efforts on better informing women and on engaging them in discussions around their values, expectations, and preferences, and how these affect their choices rather than expecting them to make firm decisions in advance of an unpredictable event as birth [36]. In addition, clients in that study stated that they wanted to wait and see before deciding on pain relief, because they lacked knowledge on pain relief options, on how painful birth would be, and how various forms of pain relief would affect their control. Another study observed significantly lower SDM scores during decisions that are postponed until the birth, compared to perinatal care [37]. Therefore, it is stressed that discussions about pain relief and other decision around birth with clients during the course of a pregnancy are important to determine each woman’s views on for example birth pain [38], to prepare for the possibility that their desired form of pain relief may not materialise [39], and engaging in discussions may ease worries as clients come close to giving birth [36].

Professionals reported several concrete needs to help low-HL clients understand their options in maternity care, including informational materials adapted to HL levels of clients. Previous literature also acknowledges the need for midwives to adapt current printed materials to meet the diverse HL levels of patients [17]. Other strategies to improve HL-sensitive communication emphasised in the literature are: the teach-back method; jargon free communication; slowing down the rate of speech; using short sentences; limiting provided information to a maximum of three main points when possible, and using patient navigators [40].

It is noteworthy that professionals’ needs for support in our study again put the emphasis on explaining options rather than supporting value clarification among clients. Professionals might also benefit from decision aids to support value clarification as part of SDM [41]. However, only a few decision aids are suitable for low-literate or low-HL clients in maternity care [41]. Also, midwives are often concerned about their own relative lack of skills and institutional support for the use of new communication technologies (e.g. social media, mobile phone applications) in antenatal care [17].

### Strengths and limitations

An important strength of this study was the use of complementary research methods including an objective assessment of SDM practices through observations, and obtaining insights from professionals themselves through a survey and interviews. Despite this strength, some methodological aspects should also be discussed. Considering the simulated research design of the observations, a learning curve might be expected during the second consultation. Nevertheless, no substantial differences were found between the first and second conversation. Also, due to COVID-19, the simulated consultations with midwives were held online, which hampered reflection on actual practices. Another limitation was the small number of respondents, low response rate in the survey, and the skewed distribution of respondents in the survey (53% obstetricians). This could only partly be explained by the length of the survey and may limit the generalizability of study findings. Furthermore, the missing values on work experience in years could hampered to gain further insights into responses in the simulated consultations and interviews, since years of experience might have impacted these responses.

### Practice implications and future research

A first step in improving SDM in the maternity care context could focus on highlighting that SDM goes beyond informing clients about options, i.e. through training. Training could support professionals in helping clients to construct provisional decisions antenatally and cope with possible deviating decisions in the run-up to actually giving birth. A previous study on HL-sensitive communication showed that training enhances professionals’ perceived skills in addressing patients’ functional, interactive, and critical HL level [42]. Future research could focus on developing interventions such as tertiary education and the effectiveness of such interventions on changes in professionals’ behaviour in daily practice as well as on the patient level. In addition, more research is needed that develops and includes the use of decision aids specific to maternity care settings that are HL-proof and clearly support information comprehension, enabling more time to be devoted to value clarification and deliberation.

## Conclusion

This study provides first insights into HL-sensitive SDM in maternity care objectively and from professionals’ point of view. Professionals’ perceived challenges and self-reported needs for support in HL-sensitive SDM were mainly focused on improving comprehension of information among clients, rather than supporting the process of value clarification and making provisional decisions together. Nevertheless, considering the explorative nature of the studies and small sample sizes, conclusions should be interpreted with caution.

### Supplementary Information

**Additional file 1.** Survey.

**Additional file 2.** Observation protocol.

**Additional file 3.** Interrater agreement in the calibration phase.

## Data Availability

The data presented in this study are available on request from the corresponding author, provided that approval from the research group is obtained and agreement is made on data security, data transfer, period of data availability, and possible use of data (for commercial ends), costs for use of data and methodology. The data are not publicly available due to privacy of participants.

## Abbreviations

SDM: Shared decision-making
HL: Health literacy
ICC: Intraclass correlation

## Footnotes

1 Interrater agreement is considered good when Intra Class Correlations (ICC) and the average weighted Kappa across items are between 0.6 and 0.8, and the raters do not differ by more than one point in their scores across items and consultations [26]. Landis JR, Koch GG. The measurement of observer agreement for categorical data. biometrics. 1977:159–74.
