# Supplementary material for: Health literate-sensitive shared decision-making in maternity care: needs for support among maternity care professionals in the Netherlands
Source: BMC Pregnancy Childbirth. 2023 Aug 21;23:594. doi: 10.1186/s12884-023-05915-9 (PMC10440871; doi:10.1186/s12884-023-05915-9)
Supplement: Supplementary file 1 — Additional file 1. Survey. [file 12884_2023_5915_MOESM1_ESM.docx]

**Supplementary file 1: survey**

1. **Background information**
2. Could you please select your sex?

- Male
- Female
- Prefer not to answer
- Other [type]

1. What is your age?

________________________________________

1. What is your function?

- Community midwife
- Locum midwife
- Hospital midwife
- Gynaecologist
- Obstetrics & Gynaecology resident (not in training)
- Nurse
- Nurse specialist
- Youth health care physician
- Youth health care nurse
- Maternity assistant
- Paediatrician
- GP
- Other, namely_____

1. How long have you been working in this function?

_______________________________________(in years)

1. **Shared Decision-Making about pain relief during birth**

We would like to know what your experiences are with Shared Decision-Making concerning pain relief options during birth.

1. When making your choice, how often do you apply Shared Decision-Making* in your communication with your client?
never – seldom - sometimes – regularly – often - always – not applicable, because I do not discuss this case with my clients (redirect to section 3)
*conversation in the consulting room, delivery room or at the hospital bed

2. Where do you experience the most problems in applying Shared Decision-Making concerning pain relief during birth? Please arrange them in order, from most difficult to least difficult.

- estimating the prior knowledge of the client

- engaging in conversation about the pros and cons of the options

- providing space to discuss the client’s preferences, questions, and insecurities

- getting a clear picture of the client’s preferences

- coming to a decision in collaboration with the client

3. When you experience problems, what do you think causes those problems?

_____________________________________________________________________

4. Which tools do you use when you engage in Shared Decision-Making about pain relief during brith? (more than one answer possible)

- training (e-learning) in Shared Decision-Making
- consultancy cards
- online decision tools
- none (redirect to 4.1)
- other, namely:
  _______________________________________________

4.1. Can you elaborate on why you do not make use of any tools during Shared Decision-Making Making about pain relief during brith?
________________________________________________________________

4.2. Can you elaborate on what you consider to be good about these tools?

________________________________________________________________

4.3 Can you elaborate on what you miss when using these tools?
________________________________________________________________

5. To what degree do you feel a need for support in Shared Decision-Making?
no need at all – not really a need – neutral – a need – a lot of need

6. In what form would you like to receive support in Shared Decision-Making? (not for ‘no need at all’)

**3. Shared Decision-Making in maternity care**

Multiple decision moments exist during the period of pregnancy and around the birth. Examples include: choosing the location of the birth, whether to use pain relief during the birth, and whether to breast- or bottle-feed. We would like to know what your experiences are with Shared Decision-Making*
*conversation in the consulting room, delivery room or at the hospital bed

1. Where do you experience the most problems applying Shared Decision-Making in general? Please arrange them in order, from most difficult to least difficult.

- estimating the prior knowledge of the client

- engaging in conversation about the pros and cons of the options

- providing space to discuss the client’s preferences, questions and insecurities

- getting a clear picture of the client’s preferences

- coming to a decision in collaboration with the client

2. When you experience problems, what do you think causes those problems?

______________________________

3. Which tools do you use when applying Shared Decision-Making in general? (more than one answer possible)

- training (e-learning) in Shared Decision-Making
- consultancy cards
- online decision tools
- none 🡪 4.1
- other, namely:
  _______________________________________________

4.1 Can you elaborate on why you do not make use of any tools when applying Shared Decision-Making in general?
__________________________________________________________________________

4.2. Can you elaborate on what you consider to be good about these tools?

4.3 Can you elaborate on what you miss when using these tools?
________________________________________________________________

5. To what degree do you feel a need for support with Shared Decision-Making in general?
no need at all – not really a need – neutral – a need – a lot of need

6. In what form would you like to receive support in applying Shared Decision-Making in general? (not for ‘no need at all’)

In our project, we would like to develop a tool that can also be used with clients with limited health literacy skills. Clients with limited health literacy skills often experience difficulties in implementing Shared Decision-Making. For example, they experience problems in communicating with their care provider, asking questions, understanding information, and applying this in their own specific situation.

7. Where do you experience the most problems when using Shared Decision-Making with clients with low health literacy skills? Please arrange them in order, from most difficult to least difficult.

- estimating the prior knowledge of the client

- engaging in conversation about the pros and cons of the options

- estimating whether the client has understood the information

- providing space to discuss the client’s preferences, questions and insecurities

- getting a clear picture of the client’s preferences

- coming to a decision in collaboration with the client

7.1 What other problems do you experience when using Shared Decision-Making with clients with low health literacy skills?
______________________________________________________________________
7.2 When you experience problems, what do you think causes those problems?

________________________________________________________________________


8. Which strategies/tools do you use when applying Shared Decision-Making with clients with low health literacy skills? (more than one answer possible)

- teach-back method
- visualisations (films, images, drawings)
- adapting the information to the level of the client
- online decision tools
- none
- other, namely:
  _______________________________________________

8.1 Can you elaborate on why do not make use of any tools when applying Shared Decision-Making with clients with low health literacy skills?
_______________________________________________________________

8.2. Can you elaborate on what you think is good about these tools?

________________________________________________________________

8.3 Can you elaborate on what you miss when using these tools?
________________________________________________________________

9. To what degree do you feel a need for support in applying Shared Decision-Making with clients with low health literacy skills?
no need at all – not really a need – neutral – a need – a lot of need

10. In what form would you like to receive support in applying Shared Decision-Making with clients with low health literacy skills (not for ‘no need at all’)

**4. In conclusion**

1. Do you have any other ideas concerning Shared Decision-Making that you would like to share with us?

__________________________________________________

Thank you for your answers. We will be organising an online co-creation session (lasting 90 minutes), in which we, in collaboration with care providers, want to think about a tool that supports clients and care providers in Shared Decision-Making.

Would you like to take part in this online co-creation session?
yes/no

(if ‘yes’, allow the possibility of leaving an e-mail address)
