# Supplementary material for: Health literate-sensitive shared decision-making in maternity care: needs for support among maternity care professionals in the Netherlands
Source: BMC Pregnancy Childbirth. 2023 Aug 21;23:594. doi: 10.1186/s12884-023-05915-9 (PMC10440871; doi:10.1186/s12884-023-05915-9)
Supplement: Supplementary file 2 — Additional file 2. Observation protocol. [file 12884_2023_5915_MOESM2_ESM.docx]

**Supplementary file 2: observation protocol**

*Introducing choice and options*

|  | **0= no effort** | **1= minimal effort** | **2= moderate effort** | **3= skilled effort** | **4= exemplary effort** | **Comments** |
| --- | --- | --- | --- | --- | --- | --- |
| The professional draws attention to or confirms​ that alternate treatment or management options exist or that the need for a decision exists.^1^ |  |  |  |  |  |  |
| The professional reassures the patient or re-affirms that the professional will​ support the patient to become informed or deliberate​ about the options.^1^ |  |  |  |  |  |  |

*Information about options*

|  | **0= no effort** | **1= minimal effort** | **2= moderate effort** | **3= skilled effort** | **4= exemplary effort** | **Comments** |
| --- | --- | --- | --- | --- | --- | --- |
| The professional gives information or checks understanding about the options that are considered reasonable (this can include taking ‘no action’), to support the patient in comparing alternatives.^1^ |  |  |  |  |  |  |

*Health literate-sensitive communication*

|  | **Not done at all** | **Done** | **Done well** | **N/a*** | **Comments** |
| --- | --- | --- | --- | --- | --- |
| The professional uses “chunk and check” (Explain 1-2 thoughts and then ask a teach back question, rather than using teach back at the end).^2^ |  |  |  |  |  |
| The professional uses plain language.^2^ |  |  |  |  |  |
| The professional supports to explanation and learning (websites, pencil and paper, models, reader-friendly print materials, non-verbal acts). ^2^ |  |  |  |  |  |
| The professional asks the patient to explain in their own words what they were told about. ^2^ |  |  |  |  |  |

*Sometimes, “chunking and checking” or “using a “tool” is not appropriate. This is the reason for the “N/A” column.

*Communication of outcome probabilities of options*

Does the midwife provide probability information about benefits and harms of options (including verbal labels, such as ’the chance exists that…’)?

- If ‘Yes’, please provide in the table below what risk was discussed and in which format.
- If ‘No’, please continue with the protocol.

|  | **Frequencies or percentages** | **Framing** (‘if x out of 100 clients suffer from this harm, it means that 100-x out of 100 clients do not suffer’) | **Only verbal labels** (‘very small risk’) | **Verbal label including numerical information**  (very small risk, namely smaller than 1%) |
| --- | --- | --- | --- | --- |
| **Epidural** |  |  |  |  |
| - Risk of paralysis (script) |  |  |  |  |
| - Fever |  |  |  |  |
| - Headache |  |  |  |  |
| - Risk of having an epidural again |  |  |  |  |
| - Other risk |  |  |  |  |
| **Remifentanil** |  |  |  |  |
| - Breathing difficulties |  |  |  |  |
| - Other risk |  |  |  |  |
| **Pethidine** |  |  |  |  |
| - Still experience pain |  |  |  |  |
| - Nausea and drowsiness |  |  |  |  |
| - Other risk |  |  |  |  |
| **No medical pain relief** |  |  |  |  |
| - Still experience pain |  |  |  |  |
| - Other risk |  |  |  |  |

*Eliciting preferences*

|  | **0= no effort** | **1= minimal effort** | **2= moderate effort** | **3= skilled effort** | **4= exemplary effort** | **Comments** |
| --- | --- | --- | --- | --- | --- | --- |
| The professional makes an effort to elicit the patient's preferences in response  to the options that have been described.^1^ |  |  |  |  |  |  |

*Integrating preferences*

|  | **0= no effort** | **1= minimal effort** | **2= moderate effort** | **3= skilled effort** | **4= exemplary effort** | **Comments** |
| --- | --- | --- | --- | --- | --- | --- |
| The professional makes an effort to integrate the patient’s elicited preferences  as decisions are made.^1^ |  |  |  |  |  |  |

**References**

1. Vortel, M. A., Adam, S., Port-Thompson, A. V., Friedman, J. M., Grande, S. W., & Birch, P. H. (2016). Comparing the ability of OPTION12 and OPTION5 to assess shared decision-making in genetic counselling. Patient education and counseling, 99(10), 1717-1723.
2. Anderson, Kathryn M., Sarah Leister, and Ruth De Rego. "The 5Ts for teach back: an operational definition for teach-back training." HLRP: Health Literacy Research and Practice 4.2 (2020): e94-e103.
