# Supplementary material for: Health literate-sensitive shared decision-making in maternity care: needs for support among maternity care professionals in the Netherlands
Source: BMC Pregnancy Childbirth. 2023 Aug 21;23:594. doi: 10.1186/s12884-023-05915-9 (PMC10440871; doi:10.1186/s12884-023-05915-9)
Supplement: Supplementary file 3 — Additional file 3. Interrater agreement in the calibration phase. [file 12884_2023_5915_MOESM3_ESM.docx]

**Supplementary file 3: interrater agreement in the calibration phase**

|  | **Set 1 (n= 4)** | | | **Set 2 (n= 4)** | | |
| --- | --- | --- | --- | --- | --- | --- |
|  | **% agreement** | **Kappa** | **ICC** | **% agreement** | **Kappa** | **ICC** |
| **OPTION-5** |  |  | **0.30** |  |  | **0.82** |
| Item 1 | 67% | 0.33^b^ |  | 100% | 0.75 |  |
| Item 2 | 50% | 0 |  | 33% | 0.75 |  |
| Item 3 | 50% | 0.14 |  | 100% | 1^a^ |  |
| Item 4 | 50% | -0.14 |  | 75% | 0.5 |  |
| Item 5 | 25% | 0^b^ |  | 67% | 0.87 |  |
| **HL-SDM** |  |  | **0.32** |  |  | **0.74** |
| Item 1 | 75% | 0 |  | 50% | 0.2 |  |
| Item 2 | 25% | 0.4^b^ |  | 100% | 1 |  |
| Item 3 | 50% | 0.33^b^ |  | 100% | 1 |  |
| Item 5 | 100% | 1^a^ |  | 100% | 1^a^ |  |

^a^ Prevalence adjusted kappa. The number of zeros in the matrix prevented calculation of kappa, while agreement was 100%. Scores were distributed across the diagonal.
^b^ Prevalence adjusted kappa. The calculation of kappa as chance agreement across 4 cells was higher than observed agreement. As this calculation inappropriately disregards the other 12 cells, we moved the 3 disagreement scores one cell up on the diagonal.(1)

**Reference**
1. Sim J, Wright CC. The kappa statistic in reliability studies: use, interpretation, and sample size requirements. Physical therapy. 2005;85(3):257-68.
